# Patterns of US Mental Health–Related Emergency Department Visits During the COVID-19 Pandemic

**DOI:** 10.1001/jamanetworkopen.2023.22720

**Published:** 2023-07-11

**Authors:** Sofia Villas-Boas, Scott Kaplan, Justin S. White, Renee Y. Hsia

**Affiliations:** 1 Department of Agricultural and Resource Economics, University of California, Berkeley; 2 Department of Economics, Naval Academy, Annapolis, Maryland; 3 Department of Epidemiology and Biostatistics, Philip R. Lee Institute for Health Policy Studies, University of California, San Francisco; 4 Department of Emergency Medicine, Philip R. Lee Institute for Health Policy Studies, University of California, San Francisco

## Abstract

**Importance:**

Numerous studies have shown that the prevalence of mental health (MH) conditions worsened during the COVID-19 pandemic. Further research is needed on this phenomenon over a longer time horizon that considers the increasing trend in MH conditions before the pandemic, after the pandemic onset, and after vaccine availability in 2021.

**Objective:**

To track how patients sought help in emergency departments (EDs) for non-MH and MH conditions during the pandemic.

**Design, Setting, and Participants:**

This cross-sectional study used administrative data on weekly ED visits and a subset of visits for MH from the National Syndromic Surveillance Program from January 1, 2019, to December 31, 2021. Data were reported from the 10 US Department of Health and Human Services (HHS) regions (Boston, New York, Philadelphia, Atlanta, Chicago, Dallas, Kansas City, Denver, San Francisco, and Seattle) for five 11-week periods. Data analysis was performed in April 2023.

**Main Outcomes and Measures:**

Weekly trends in total ED visits, mean MH-related ED visits, and proportion of ED visits for MH conditions were investigated to determine changes in each measure after the pandemic onset. Prepandemic baseline levels were established from 2019 data, and time trends of these patterns were examined in the corresponding weeks of 2020 and 2021. A fixed-effects estimation approach with weekly ED region data by year was used.

**Results:**

There were 1570 total observations in this study (52 weeks in 2019, 53 weeks in 2020, and 52 weeks in 2021). Statistically significant changes in non-MH and MH-related ED visits were observed across the 10 HHS regions. The mean total number of ED visits decreased by 45 117 (95% CI, −67 499 to −22 735) visits per region per week (39% decrease; *P* = .003) in the weeks after the pandemic onset compared with corresponding weeks in 2019. The mean number of ED visits for MH conditions (−1938 [95% CI, −2889 to −987]; *P* = .003) decreased significantly less (23% decrease) than the mean number of total visits after the onset of the pandemic, increasing the mean (SD) proportion of MH-related ED visits from 8% (1%) in 2019 to 9% (2%) in 2020. In 2021, the mean (SD) proportion decreased to 7% (2%), and the mean number of total ED visits rebounded more than that of mean MH-related ED visits.

**Conclusions and Relevance:**

In this study, MH-related ED visits demonstrated less elasticity than non-MH visits during the pandemic. These findings highlight the importance of addressing the provision of adequate MH services, both in acute and outpatient settings.

## Key Points

**Question:**

Did patterns of US emergency department (ED) visits and mental health (MH)–related ED visits change during the COVID-19 pandemic?

**Findings:**

In this cross-sectional study with 1570 total observations for January 1, 2019, to December 31, 2021, mean total ED visits decreased more than the proportion of ED visits for MH conditions during the weeks after the pandemic was declared in 2020 compared with the corresponding weeks in 2019. The mean proportion of MH-related ED visits increased from 8% in 2019 to 9% in 2020 and then decreased to 7% in 2021, when mean total ED visits rebounded more than MH-related visits.

**Meaning:**

The finding that ED visits for MH decreased less than visits for other reasons during the pandemic suggests that patients with non-MH conditions may have greater flexibility during public health emergencies, whereas there may be fewer alternatives for MH care.

## Teaser

This cross-sectional study examines weekly patterns of emergency department (ED) visits and mental health–related ED visits for 10 US regions during the COVID-19 pandemic.

## Introduction

Numerous studies report that adverse mental and behavioral health conditions, including depression, anxiety, substance use, and suicidal ideation, became more prevalent in the early months of the COVID-19 pandemic in 2020 and into 2021 compared with prior years.^1,2,3,4^ Even before the pandemic onset, literature reported an increased demand for psychiatric care, as measured by emergency department (ED) visits for mental health (MH) conditions.^5,6^ These changes in MH status were prevalent not only in the US but also in many other countries. According to a World Health Organization report, the global prevalence of anxiety and depression increased by 25% during the first year of the pandemic.^7^ Other research also showed a 28% increase in major depressive disorder and a 25% increase in anxiety disorders in 2020.^8^ One study reported that rates of major depressive disorder and acute stress or posttraumatic stress disorder within the first few weeks of the pandemic in the US were approximately 3 times higher and rates of generalized anxiety disorder were 10 to 16 times higher compared with prepandemic levels.^9^ The prevalence of MH conditions continued to worsen into 2021, as evidenced by a US Centers for Disease Control and Prevention (CDC) report stating that the percentage of adults with recent symptoms of an anxiety or depressive disorder increased from 36.4% in August 2020 to 41.5% in February 2021.^10^

Less is known regarding patterns in ED visits for MH conditions during the COVID-19 pandemic. A widely cited study on ED visit trends in the US during the pandemic reported that the total number of ED visits for any cause decreased by 42% from March 2019 to March 2020.^11^ While the number of ED visits specifically for MH conditions also declined in March and April 2020, one repeated cross-sectional study of approximately 190 million ED visits reported that proportions of visits for MH conditions, suicide attempts, all drug and opioid overdoses, intimate partner violence, and child abuse and neglect were higher in mid-March through October 2020 compared with the same period in 2019.^12^ Additional studies have reported general trends in MH during the pandemic, but they lack prepandemic comparison periods to infer whether more recent changes were driven by the pandemic or were an extension of secular trends.^8,11^ Other studies include data up until 2020 but do not include 2021.^5,6,7^ Our study aimed to extend these findings and use the most current longitudinal regional data at the weekly level to examine general trends in ED use related to COVID-19 and quantify how ED visits for MH changed from 2019 to 2021.

## Methods

### Data Source

This cross-sectional study used the CDC National Syndromic Surveillance Program (NSSP) Mental Health ED visit data set, version 1, which included ED visit–level information by region and week for the 10 US Department of Health and Human Services (HHS) regions (Boston, New York, Philadelphia, Atlanta, Chicago, Dallas, Kansas City, Denver, San Francisco, and Seattle) for January 1, 2019, through December 31, 2021 (eAppendix 1 in Supplement 1). The data set is based on administrative data reported by local and state public health departments as part of the NSSP^13^ and is a convenience sample with ED visit data from 71% of US facilities. The unit of observation is denoted at the week level by HHS region. The University of California, Berkeley Institutional Review Board deemed this study as not human participant research and waived informed consent. The study followed the Strengthening the Reporting of Observational Studies in Epidemiology (STROBE) reporting guideline.

### Definition of MH-Related ED Visits

Mental health–related ED visits were identified within the NSSP data set using the CDC Mental Health query, version 1, which included visits (outpatient and admitted) in which there were acute MH crises (ie, the sole or primary reason for the visit was only related to MH) and visits in which MH conditions were present (defined as coded in the discharge diagnosis or mentioned in the chief concern text) but may not have been the sole reason for the visit. The complete list of chief concerns and diagnosis codes used to classify visits by MH syndrome is presented in eTable 1 in Supplement 1.

### Statistical Analysis

We used longitudinal weekly regional data for 2019, 2020, and 2021 to assess 3 outcomes: total weekly number of ED visits, number of weekly MH-related ED visits, and proportion of ED visits due to MH conditions. Data were aggregated to the week level for all age cohorts. We established baseline levels from 2019, and then examined time trends of these patterns during the corresponding weeks of 2020 and 2021. We used a fixed-effects estimation approach with weekly ED region data by year. Given that the pandemic was declared in week 12 of 2020, we defined the following five 11-week period indicators: WP1, weeks 0 to 11 (equal to 1 for the first 11 weeks for all years and 0 otherwise); WP2, weeks 12 to 23; WP3, weeks 24 to 35; WP4, weeks 36 to 47; and WP5, weeks 48 to 53 in 2020 and weeks 48 to 52 in 2021.

In separate regressions for each of the 3 outcomes, we denoted Y_rwy_ the linear model for geographic region r (Region_r_), year t (Year_t_), and week of year w (Week_w_) as follows: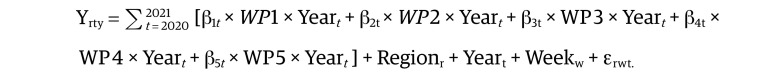.Region_r_ was a vector of region fixed effects for each region, Year_t_ was a set of indicators for each year (2019 was the omitted year), and Week_w_ was a vector of week-of-year fixed effects that controlled for week-of-year changes in outcomes common to all regions and measured relative to the baseline week of year (defined as the first week of 2019). These week-of-year fixed effects common to all regions were included in an attempt to capture outcomes associated with seasonality at the week level common to all regions. Finally, ε_rwt_ was an idiosyncratic error term composed of unobserved determinants of each outcome that were not controlled for by the variables specified in the linear model in the Equation.

The coefficients of interest were associated with the interactions between each of the 11-week period indicators (WP1-WP5) and the 2020 and 2021 indicator variables. β_1_ coefficients measured changes in outcomes during weeks 1 to 11 (ie, January until mid-March) in 2020 and separately in 2021, relative to the same weeks in 2019. β_2_ coefficients measured changes in outcomes during the following 11 weeks in 2020 and separately in 2021, relative to the same weeks in 2019. Similarly, β_3,_ β_4_, and β_5_ measured changes in outcomes in the corresponding weeks in 2020 and 2021 (separately) relative to the corresponding weeks in 2019.

We used the Equation to estimate values separately for the total number of ED visits, the number of MH-related ED visits, and the proportion of ED visits due to MH conditions by week. Our model allowed us to identify how these outcomes changed in 2020 and 2021 (separately) relative to the baseline corresponding weeks in 2019. We did this while explicitly controlling for other confounding factors specific to each week, region, and year. Finally, robust SEs were clustered by region to account for correlated outcomes over time within a region.

Analyses were conducted using Stata, version 14.2 (StataCorp), with 2-sided *P* < .05 considered statistically significant. Data analysis was performed in April 2023.

## Results

### Summary Statistics

There were 1570 total observations of ED visits per week in the 10 HHS regions from January 2019 to December 2021 (52 weeks in 2019, 53 weeks in 2020, and 52 weeks in 2021). Table 1 presents summary statistics and annual breakdowns for 2019, 2020, and 2021 for the key outcomes of interest averaged across all 10 HHS regions. The unit of observation was a specific week in a specific region (week-region). The mean (SD) number of weekly ED visits per HHS region was 126 117 (96 462; range, 12 598-455 572). On average, the mean (SD) number of ED visits per week-region for MH conditions was 9374 (6751; range, 1068-29 169). The mean proportion of ED visits for MH conditions was 8% (range, 3%-12%).

**Table 1.** Summary Statistics of Total ED Visits, MH-Related ED Visits, and Proportion of MH-Related ED Visits, 2019 to 2021^a^

Year	Mean (SD) No. of ED visits [range]
All (N = 1570)	
Total	126 117 (96 462) [12 598-455 572]
MH related	9374 (6751) [1068-29 169]
Ratio of MH related/total	0.08 (0.02) [0.03-0.12]
2019 (n = 520)	
Total	135 260 (101 009) [17 099-399 915]
MH related	9886 (7076) [1513-28 920]
Ratio of MH related/total	0.08 (0.01) [0.03-0.11]
2020 (n = 530)	
Total	115 277 (90 512) [12 598-416 364]
MH related	9337 (6917) [1068-29 169]
Ratio of MH related/total	0.09 (0.02) [0.04-0.12]
2021 (n = 520)	
Total	128 022 (96 796) [15 956-455 572]
MH related	8901 (6201) [1258-25 566]
Ratio of MH related/total	0.07 (0.02) [0.03-0.12]

Abbreviations: ED, emergency department; MH, mental health.

^a^ The unit of observation is a specific week in a specific US Department of Health and Human Services region. There were 10 regions for 52 weeks in 2019, 53 weeks in 2020, and 52 weeks in 2021, resulting in a total of 1570 observations. Total ED visits, MH-related ED visits, and the percentage of ED visits for MH conditions were obtained from the National Syndromic Surveillance Program data set.

In our comparison of the 3 study years, we observed that the mean number of total ED visits and MH-related ED visits decreased in 2020 relative to 2019. Since the mean number of total ED visits decreased more than the mean number of MH-related ED visits, the mean (SD) proportion of ED visits for MH conditions increased from 8% (1%) in 2019 to 9% (2%) in 2020. During the pandemic, this proportion reached a maximum of 12% in the Seattle region in April 2020, whereas the maximum was 11% in the Kansas City region in March 2019. In 2021, the mean (SD) proportion decreased to 7% (2%), primarily due to the mean number of total ED visits rebounding more than that of MH-related ED visits.

### Weekly Patterns

The Figure depicts weekly time trends over the 10 HHS regions for total ED visits, MH-related ED visits, and proportion of ED visits due to MH conditions by week. Total ED visits had a comparable stable trend for 2019 and 2020 during the weeks leading up to the second week of March (Figure, A). The mean (SD) number of weekly ED visits for each region was 135 260 (101 009) in 2019, which decreased to 115 277 (90 512) in 2020 once the global pandemic was declared (Table 1). The number of total ED visits in 2021 started lower than in 2020 and 2019 but eventually returned to 2019 levels during weeks 24 to 35, resulting in a mean (SD) of 128 022 (96 796) visits per week-region in 2021.

**Figure.**
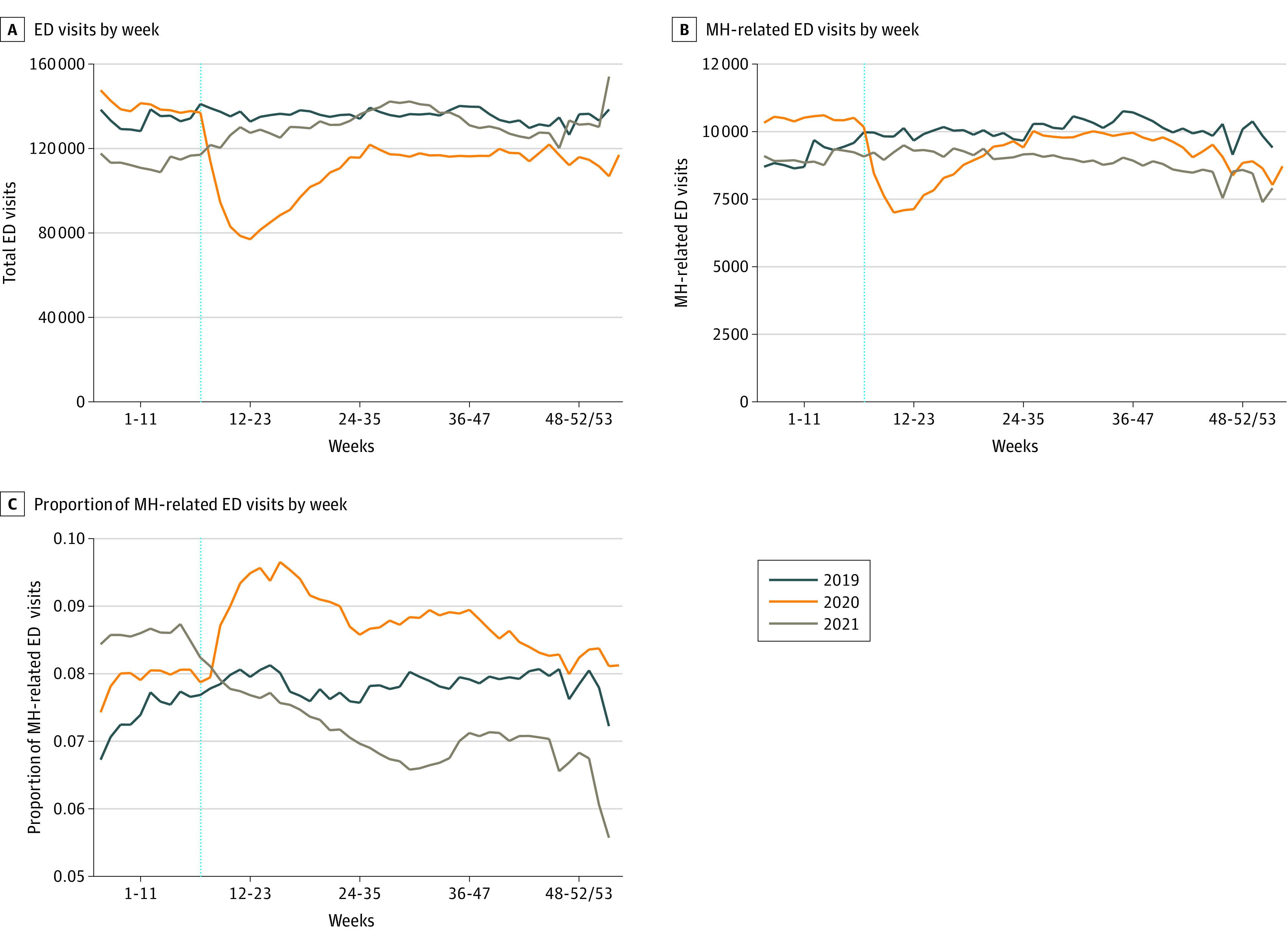
Patterns of Emergency Department (ED) Visits and Mental Health (MH)–Related ED Visits Across 10 US Department of Health and Human Services Regions in 2019, 2020, and 2021 A, Total number of ED visits. B, Number of MH-related ED visits. C, Proportion of ED visits for MH conditions.

The absolute number of MH-related ED visits increased slightly until mid-March in 2019, while the number of MH-related visits over the same period in 2020 was higher than in 2019 and fairly stable (Figure, B). Once the global pandemic was declared in 2020, the total number of MH-related ED visits decreased sharply compared with the same weeks in 2019. While the total number of MH-related ED visits increased later in summer 2020, it remained lower than that in 2019 for similar weeks. The mean (SD) number of weekly MH-related ED visits was 9886 (7076) in 2019, which decreased to 9337 (6917) in 2020 (Table 1). The total number of MH-related ED visits in 2021 began at a lower level than in 2020 and was comparable with the 2019 level. The number of MH visits remained fairly stable throughout 2021 at levels lower than in 2019 during the same weeks. The mean (SD) total number of MH-related ED visits per week-region was 8901 (6201) in 2021.

Finally, the Figure, C, depicts the proportion of MH-related ED visits in each of the 3 years studied. The 2019 and 2020 trends were roughly parallel until the global pandemic onset in 2020. Thereafter, the proportion of MH-related ED visits increased in 2020 compared with the same weeks in 2019 and remained higher than in 2019 for similar weeks. The proportion of MH-related ED visits in 2021 returned to similar weekly patterns observed in early 2019 and then continued to decrease during the second half of 2021 relative to corresponding weeks in 2019. As shown in Table 1, the mean (SD) proportion of ED visits for MH conditions was 8% (1%) in 2019, increased to 9% (2%) in 2020, and decreased to 7% (2%) in 2021.

### Regression Estimates

Table 2 presents the estimated total number of ED visits, the number of MH-related ED visits, and the proportion of MH-related ED visits (calculated with the Equation). Robust SEs clustered by region are shown in parentheses in Table 2. The first column examines the number of MH-related ED visits by week-region. The estimates from weeks 1 to 11 in 2020 (January until mid-March 2020) correspond to the weeks just before COVID-19 was declared a global pandemic and before any public health mandates were implemented. During this time, MH-related visits were significantly higher than in the same months of 2019 by a total of 1264.3 weekly visits per region (95% CI, 613.1 to 1915.1; *P* = .004).

**Table 2.** Regression Estimates of Change in Outcomes in Weeks of 2020 and 2021 Relative to the Same Weeks in 2019

Week	Change relative to 2019 (N = 1570)^a^					
	No. of MH-related ED visits	*P* value	Total No. of ED visits	*P* value	Proportion of MH-related ED visits	*P* value
2020						
1-11	1264.3 (325.4)	.004	5569.4 (1911.0)	.02	0.005 (0.002)	.06
12-23	−1938.0 (475.3)	.003	−45 116.6 (11 191.0)	.003	0.013 (0.004)	.01
24-35	−362.8 (296.0)	.25	−19 934.1 (4469.3)	.002	0.010 (0.003)	.01
36-47	−681.4 (350.7)	.08	−17 636.8 (3991.2)	.002	0.006 (0.003)	.06
48-53	−1216.3 (398.9)	.01	−21 951.8 (5793.4)	.004	0.005 (0.003)	.15
2021						
1-11	−157.5 (255.4)	.55	−20 458.1 (5230.7)	.01	0.011 (0.003)	.01
12-23	−713.6 (358.9)	.08	−8924.8 (2589.4)	.01	−0.002 (0.004)	.54
24-35	−1112.3 (421.6)	.03	1727.2 (2822.5)	.56	−0.010 (0.003)	.01
36-47	−1630.9 (488.8)	.01	−6211.7 (2423.8)	.03	−0.009 (0.002)	.003
48-53	−1603.3 (448.2)	.01	1917.8 (1204.8)	.15	−0.013 (0.002)	.000
*R* ^2^	0.99	NA	0.99	NA	0.85	NA

Abbreviations: ED, emergency department; MH, mental health; NA, not applicable.

^a^ Robust SEs clustered by region are given in parentheses. All coefficients are relative to the corresponding weeks in 2019. For example, the value for weeks 1 to 11 in 2020 is the mean for weeks 1 to 11 in 2020 relative to the same weeks in 2019. These specifications control for region fixed effects, year fixed effects, and week fixed effects, and the baseline year is 2019.

Just after the pandemic onset in 2020, ED visits due to MH decreased significantly (−1938.0 [95% CI, −2888.6 to −987.4]; *P* = .003) relative to the same 11-week period in 2019. Changes in MH-related visits in two 11-week periods (weeks 24-35 and 36-47) in 2020 relative to the same period in 2019 were not statistically significant. In the last weeks of 2020 (weeks 48-53), the number of MH-related visits was significantly lower than that in 2019 (−1216.3 [95% CI, −2014.1 to −418.5]; *P* = .01).

During the first weeks of 2021, MH-related ED visits returned to similar levels, on average, as during the corresponding weeks of 2019. During weeks 12 to 23 of 2021, the mean number of MH-related ED visits was lower than in the corresponding weeks of 2019, though this difference was not significant, and MH-related ED visits were higher than in 2020. In the remaining weeks of 2021, MH-related ED visits were, on average, lower than in the corresponding weeks of 2019, although they rebounded to a higher level than during those same weeks in 2020.

Table 2 provides estimates of changes in the total number of ED visits by week-region. Although the first 11 weeks of 2020 had more ED visits than the corresponding months in 2019 (5569.4 [95% CI, 1747.4 to 9391.4]; *P* = .02), total ED visits decreased significantly during the period immediately after the 2020 pandemic declaration relative to the same period in 2019 (−45 116.6 [95% CI, −67 498.6 to −22 734.6]; *P* = .003) and total ED visits remained significantly lower during the remaining weeks in 2020 relative to 2019. In a comparison of the first weeks in 2021 to the same weeks in 2019, there were significantly fewer ED visits (−20 458.1 [95% CI, −30 919.5 to −9996.7]; *P* = .01), on average. This trend continued for all remaining weeks in 2021 except for weeks 24 to 35, in which ED visits did not change significantly compared with 2019 (1727.2 [95% CI, −3917.8 to 7372.2]; *P* = .56).

Finally, Table 2 also provides estimates of changes over time in the proportion of total MH-related ED visits by week and by region. During weeks 12 to 23 after the pandemic declaration in 2020, the proportion of MH-related ED visits increased significantly relative to the mean during the same weeks in 2019 (1.3 percentage points [95% CI, 0.6 to 2.0 percentage points]; *P* = .01). This number remained significantly higher during weeks 24 to 35 in 2020 relative to 2019 (1.0 percentage point [95% CI, 0.4 to 1.7 percentage points]; *P* = .01) and was not significantly different for the remaining weeks in 2020 relative to 2019. In the first weeks of 2021, there was a higher proportion of MH-related ED visits than during the corresponding weeks in 2019 (1.1 percentage points [95% CI, 0.4 to 1.7 percentage points]; *P* = .01). Beginning in weeks 24 to 35, there was a lower proportion of MH-related ED visits than in the corresponding weeks of 2019 (−1.0 percentage point [95% CI, −1.5 to −0.3 percentage points]; *P* = .01) and the proportion of MH-related ED visits remained significantly lower during the remaining weeks of 2021 compared with 2019.

These estimates suggest that immediately after the pandemic declaration (weeks 12-23) in 2020, the mean (SE) number of MH-related ED visits decreased significantly by 1938.0 (475.3) weekly visits by region (23% decrease), relative to the same weeks in 2019 (eTable 2 and eAppendix 2 in Supplement 1). In terms of total ED visits, there was a 39% decrease (representing <45 116 visits per week-region) in weeks 12 to 23 of 2020 relative to 2019 (eTable 2 and eAppendix 2 in Supplement 1). Although total ED visits rebounded later in the pandemic in 2021, MH-related visits did not return to their levels in corresponding weeks of 2019.

## Discussion

Using longitudinal data on weekly ED visits in the 10 HHS regions from January 2019 to December 2021, this study reports temporal patterns in which patients sought help in the ED for both non-MH and MH reasons before and during the COVID-19 pandemic. We found that total ED and MH-related ED visits decreased significantly in March 2020 after COVID-19 was declared a global pandemic compared with spring 2019. Specifically, total ED visits decreased by 39% during weeks 12 to 23 of 2020 relative to 2019. However, in 2021, total ED visits returned to levels higher than those observed in 2019. The proportion of MH-related ED visits decreased by 23% in weeks 12 to 23 of 2020, and had not returned to levels observed in corresponding weeks of 2019 as of December 2021. The shorter duration and intensity of the decrease in total ED visits resulting from the pandemic led to an increase in the proportion of MH-related ED visits. Second, we found that the mean proportion of MH-related ED visits increased from 8% in 2019 to 9% in 2020 during the height of the pandemic and then decreased in 2021 to 7%.

Our findings are consistent with other reports showing a rapid decline in overall ED visits in March and April 2020. A widely cited study from the CDC on ED visit trends in the US during the pandemic reported that the total number of ED visits in the US decreased by 42%, from approximately 2 099 734 visits per week in March and April 2019 to about 1 220 211 per week in March and April 2020.^11^ The lowest number of visits reported between January 2019 and May 2020 was from April 12 to 18, 2020. In another study, the results showed that the number of ED visits for MH conditions also decreased in March and April 2020; however, a cross-sectional study of almost 190 million ED visits found that visit rates for MH conditions, suicide attempts, all drug and opioid overdoses, intimate partner violence, and child abuse and neglect were higher in mid-March through October 2020 during the COVID-19 pandemic compared with the same period in 2019.^12^ The number of MH-related ED visits was also higher after COVID-19 surges, specifically for young adults and individual racial and ethnic minority subpopulations.^13^ Our findings extend this prior literature by analyzing additional years of data and suggest that MH-related ED visits on both an absolute and relative scale, as a proportion of all ED visits, did not continue to increase through 2021.

There are several important implications associated with these patterns. First, the increased proportion of MH-related ED visits may be interpreted as an increase in the underlying need for critical care MH services. As suggested by the literature, patients with more severe illness tended to present to the ED during the pandemic, resulting in a larger proportion of higher-acuity and admitted visits compared with nonpandemic times.^9^ Further, this decrease in lower-acuity visits (eg, for sprains, strains, or chronic conditions) typically observed during nonpandemic times may have constrained the number of MH-related visits observed because of long ED wait times, resulting in an underestimate of critical MH needs before the pandemic.

Although total ED visits decreased sharply with the pandemic onset in 2020 and rebounded quickly to prepandemic levels in 2021, MH-related visits were less elastic and did not return to prepandemic levels. This finding suggests that patients with non-MH conditions may have a greater ability to adapt to public health emergencies, such as a global pandemic, than those with MH conditions. While patients seeking care for certain non-MH issues may have the flexibility to delay treatment or pursue alternative options for care, patients seeking MH services may have fewer options.

### Limitations

Our study has a few important limitations related to data quality. First, the classification of near real-time feeds of ED encounters involves text and diagnostic code processing to standardize unstructured fields. Algorithms look for key terms and diagnostic codes to create syndrome categories automatically as new data are processed. As a result, misclassification of encounters is possible during the syndrome classification process. Second, the NSSP ED surveillance data are not nationally representative; they include ED visits from 71% of US facilities, and facility participation varies within and across states as well as over time. To minimize data quality shifts over time, the data set obtained from the CDC was restricted to facilities reporting consistently throughout the sample period and to those with more complete data, with an average weekly ability to classify discharge diagnosis informativeness 70% of the time or more throughout the study period. Finally, the data do not allow us to distinguish whether the primary visit cause was related to an MH condition or if the condition was discovered during the visit itself.

We note that these data likely understate the true prevalence of emergency conditions, specifically MH conditions, because persons with less severe injuries or conditions may have been less likely to seek emergency care during the pandemic when many avoided public spaces to reduce their risk for SARS-CoV-2 infection and subsequent COVID-19. As a result, our findings should be interpreted in terms of patterns of ED-seeking behavior overall and for MH conditions.

## Conclusions

The findings of this cross-sectional study illuminate the increasing importance of providing adequate MH services in both acute and outpatient settings. To adequately address MH needs, health care stakeholders and policy makers must respond to the immediate disruption caused by COVID-19 and the longstanding structural inadequacies in the US mental health care system that were exacerbated by the pandemic. This may include expanding the quantity, scope, and availability of services to improve patient adaptability, specifically in times of crisis.

## Notes

Supplement 1. **eAppendix 1.** Supporting Information on US Department of Health and Human Services (HHS) Regions **eTable 1.** Keyword Syndrome Descriptions With Free-Text Reason for Visit (Chief Concern) Search Terms and Diagnosis Codes Included in Keyword Syndromes for Emergency Department Visits Associated With Mental Health Conditions (MHCs)—National Syndromic Surveillance Program **eTable 2.** Change in Log of Mental Health (MH) Emergency Department (ED) Visits and Log of Total ED Visits in 2020 and 2021 Relative to the Corresponding Baseline Levels in 2019 **eAppendix 2.** Alternative Specification for eTable 2

Supplement 2.
Data Sharing Statement
