# Supplementary material for: Patterns of US Mental Health–Related Emergency Department Visits During the COVID-19 Pandemic
Source: JAMA Netw Open. 2023 Jul 11;6(7):e2322720. doi: 10.1001/jamanetworkopen.2023.22720 (PMC10336606; doi:10.1001/jamanetworkopen.2023.22720)
Supplement: Supplement 1. — eAppendix 1. Supporting Information on US Department of Health and Human Services (HHS) Regions eTable 1. Keyword Syndrome Descriptions With Free-Text Reason for Visit (Chief Concern) Search Terms and Diagnosis Codes Included in Keyword Syndromes for Emergency Department Visits Associated With Mental Health Conditions (MHCs)—National Syndromic Surveillance Program eTable 2. Change in Log of Mental Health (MH) Emergency Department (ED) Visits and Log of Total ED Visits in 2020 and 2021 Relative to the Corresponding Baseline Levels in 2019 eAppendix 2. Alternative Specification for eTable 2 [file jamanetwopen-e2322720-s001.pdf]

Supplemental Online Content

Villas-Boas S, Kaplan S, White JS, Hsia RY. Patterns of US mental health–related emergency department visits during the COVID-19 pandemic. *JAMA Netw Open*. 2023;6(7):e2322720. doi:10.1001/jamanetworkopen.2023.22720

eAppendix 1. Supporting Information on US Department of Health and Human Services (HHS) Regions
eTable 1. Keyword Syndrome Descriptions With Free-Text Reason for Visit (Chief Concern) Search Terms and Diagnosis Codes Included in Keyword Syndromes for Emergency Department Visits Associated With Mental Health Conditions (MHCs)—National Syndromic Surveillance Program
eTable 2. Change in Log of Mental Health (MH) Emergency Department (ED) Visits and Log of Total ED Visits in 2020 and 2021 Relative to the Corresponding Baseline Levels in 2019
eAppendix 2. Alternative Specification for eTable 2

This supplemental material has been provided by the authors to give readers additional information about their work.

© 2023 Villas-Boas S et al. *JAMA Network Open*.

eAppendix 1. Supporting Information on US Department of Health and Human Services (HHS) Regions

The 10 HHS regions are as follows:
Region 1 - Boston: Connecticut, Maine, Massachusetts, New Hampshire, Rhode Island, and Vermont.
Region 2 - New York: New Jersey, New York, Puerto Rico, and the Virgin Islands.
Region 3 - Philadelphia: Delaware, District of Columbia, Maryland, Pennsylvania, Virginia, and West Virginia.
Region 4 - Atlanta: Alabama, Florida, Georgia, Kentucky, Mississippi, North Carolina, South Carolina, and Tennessee.
Region 5 - Chicago: Illinois, Indiana, Michigan, Minnesota, Ohio, and Wisconsin.
Region 6 - Dallas: Arkansas, Louisiana, New Mexico, Oklahoma, and Texas.
Region 7 - Kansas City: Iowa, Kansas, Missouri, and Nebraska.
Region 8 - Denver: Colorado, Montana, North Dakota, South Dakota, Utah, and Wyoming.
Region 9 - San Francisco: Arizona, California, Hawaii, Nevada, American Samoa, Commonwealth of the Northern Mariana Islands, Federated States of Micronesia, Guam, Marshall Islands, and the Republic of Palau.
Region 10 - Seattle: Alaska, Idaho, Oregon, and Washington.

© 2023 Villas-Boas S et al. *JAMA Network Open*.

eTable 1. Keyword Syndrome Descriptions With Free-Text Reason for Visit (Chief Concern) Search Terms and Diagnosis Codes Included in Keyword Syndromes for Emergency Department Visits Associated With Mental Health Conditions (MHCs)—National Syndromic Surveillance Program

Definition	Chief Complaint Search Terms	Diagnosis Codes
**CDC Overall Mental Health Conditions v1**	Schizophrenia (include misspellings)<br>Schizotypal (include misspellings)<br>Schizoaffective (include misspellings)<br>Grief (include misspellings)<br>Grieve (include misspellings)<br>Bereave (include misspellings)<br>Psych<br>Mental health<br>Mental evaluation<br>Mental problem<br>Mentally<br>Mental illness<br>Reactive attachment<br>Auditory hallucination<br>Visual hallucination<br>Hearing voice<br>Evaluation hallucination<br>History of hallucination<br>Hallucinations patient<br>Hallucinations unspecified<br>Seeing thing<br>Delusion (include misspellings)<br>Paranoia (include misspellings)<br>Trichotillomania<br>Dissociative disorder<br>Dissociative conversion<br>Dissociative identity<br>Dissociative and conversion<br>Dissociative tendency<br>Dissociative episode<br>Dissociative conversion<br>Dissociative behavior<br>Dissociative amnesia<br>Dissociative attentive<br>Experiencing dissociation<br>Dissociative (when used with depress, depression, psych, dysthymic, anxiety, anxious, traumatic stress, PTSD, adjustment disorder, mental, attention deficit, bipolar, schizophrenia, schizoaffective, schizotypal – and associated misspellings of all terms)	295.00; 295.01; 295.02; 295.03; 295.04; 295.10; 295.11; 295.12; 295.13; 295.14; 295.20; 295.21; 295.22; 295.23; 295.24; 295.30; 295.31; 295.32; 295.33; 295.34; 295.40; 295.41; 295.42; 295.43; 295.44; 295.50; 295.51; 295.52; 295.53; 295.54; 295.60; 295.61; 295.62; 295.63; 295.64; 295.70; 295.71; 295.72; 295.73; 295.74; 295.80; 295.81; 295.82; 295.83; 295.84; 295.90; 295.91; 295.92; 295.93; 295.94296.90; 296.99; 297.0; 297.1; 297.2; 297.3; 297.8; 297.9; 298.0; 298.1; 298.2; 298.3; 298.4; 298.8; 298.9; 300.10; 300.11; 300.12; 300.13; 399.14; 300.15; 300.81; 300.82; 300.89; 300.7; 307.80; 307.89; 300.9 648.40; 648.41, 648.42, 648.43; 648.44; V62.82<br><br>F20; F21; F22; F23; F25; F28; F29; F39; F44; F45; F48; F53.1; F54; F63.3; F93.8; F93.9; F94; F98.9; F99; Z63.4<br><br>413307004; 74732009; 231538003; 268664001; 48500005; 191667009; 41526007; 18193002; 224965009; 74506000; 58214004; 268617001; 64905009;191526005; 68890003

© 2023 Villas-Boas S et al. *JAMA Network Open*.

**CDC Depressive Disorders v1**	Depression<br>Dysthymic<br>Dysthymic<br>Mood disorder<br>Mood disturbance	296.20; 296.21; 296.22; 296.23; 296.24; 296.25; 296.30; 296.31; 296.32; 296.33; 296.34; 296.35; 300.4; 309.0; 309.1; 296.90; 296.99; 309.28<br><br>F32 (except F32.5); F33 (except F33.42); F34.1; F34.9; F43.21; F43.23; O90.6<br><br>35489007; 310497006; 370143000; 712823008; 57194009
**CDC Attention-Deficit/Hyperactivity Disorders v1**	Attention deficit<br>Hyperactivity<br>Attention problems	314.00, 314.01; 314.9; 314.2<br><br>F90 (include all in this series)<br><br>406506008<br>35253001
**CDC Trauma and Stressor-related Disorders v1**	Traumatic stress<br>PTSD (including misspellings)<br>Adjustment disorder<br>Reaction to acute stress<br>Acute stress reaction<br>Acute stress crisis<br>Acute stress eval<br>Eval acute stress<br>Acute stress behavior (including misspellings for behavior)<br>Behavior acute stress<br>Behavior acute stress<br>Reaction to severe stress<br>Severe stress reaction<br>Severe stress crisis	308.0; 308.1; 308.2; 308.3; 308.4; 308.9; 309.81; 309.89; 309.9; 309.0; 309.1; 309.24; 309.28;309.29; 309.3; 309.4<br><br>F43<br><br>67195008; 47505003; 17226007; 57194009; 271952001
**CDC Disruptive Behavioral and Impulse-Control v1**	Conduct disorder<br>Oppositional defiant (with spelling mistakes for "defiant")<br>Antisocial personality<br>Disturbance of conduct<br>Impulse control disorder<br>Explosive disorder	309.3; 309.4; 312.00; 312.01; 312.02; 312.03; 312.10; 312.11; 312.12; 312.13; 312.20, 312.21; 312.22; 312.23; 312.4; 312.81; 312.82; 312.89; "312.9; 312.30; 312.31; 312.39; 313.81; 314.2; 301.7<br><br>F43.24; F43.25; F91.0; F91.1; F91.2; F91.3; F91.8; F91.9; F63.81; F63.1; F63.2; F63.9; F63.0; F63.89; F60.2<br><br>54319003; 18941000; 430909002; 26665006
**CDC Bipolar Disorders v1**	Bipolar<br>Manic<br>Cyclothymic<br>Mania<br>Hypomania<br>Hypermania	296.00; 296.01; 296.02; 296.03; 296.04; 296.05; 296.40; 296.41; 296.42; 296.43; 296.44; 296.45; 296.50; 296.51; 296.52; 296.53; 296.54; 296.55; 296.60; 296.61; 296.62; 296.63; 296.64; 296.65; 296.7<br>296.80; 296.81; 296.82; 296.89<br><br>F30.1; F30.2; F30.3; F30.8; F30.9; F31.0; F31.1; F31.2; F31.3; F31.4; F31.5; F31.6; F31.7 (only include: F31.70, F31.71, F31.73, F31.75, F31.77); F31.8; F31.9; F34.0<br><br>191627008; 371596008; 83225003; 231494001; 284513006; 268619003

© 2023 Villas-Boas S et al. *JAMA Network Open*.

**CDC Eating Disorders v1**	Pica<br>Bulimia (including misspellings)<br>Binge eating (including misspellings of binge)<br>Binge-eating<br>Eating disorder<br>Rumination disorder<br>Binging or purging<br>Binging and purging<br>Weight loss or anorexia<br>Anorexia nervosa (including misspellings of anorexia)<br>Anorexic (including misspellings of anorexic)<br>Being treated for anorexia (including misspellings of anorexia)<br>Treatment for (including misspellings of anorexia)<br>Weight loss (including misspellings of weight; include when used with anorexia)<br>Loss of weight (including misspellings of weight; include when used with anorexia)<br>Weight loss (including misspellings of weight; include when used with anorexia)<br>Loss of weight (including misspellings of weight; include when used with anorexia)<br>Eating disorder<br>Anorexia history (including misspellings of anorexia)<br>History of anorexia (including misspellings of anorexia)<br>Mental anorexia (including misspellings of anorexia)<br>Mental (when used with anorexia)<br>Psych (when used with anorexia)	307.1; 307.50; 307.51; 307.52; 307.53; 307.54; 307.59<br><br>F50.00; F50.01; F50.02; F50.2; F50.8; F50.82; F50.89; F50.9; F98.21; F98.29; F98.3<br><br>72366004; 56882008; 7800400; 32721004
**CDC Tic Disorders v1**	Neurological tic<br>Tic disorder<br>Behavioral tic<br>Facial tic<br>Face tic	307.20; 307.21; 307.22; 307.23<br><br>F95; G25.69<br><br>568005; 5158005
**CDC Obsessive-Compulsive Disorders v1**	Obsessive compulsive<br>Compulsive disorder	300.3<br><br>F42<br><br>191736004

© 2023 Villas-Boas S et al. *JAMA Network Open*.

eTable 2. Change in Log of Mental Health (MH) Emergency Department (ED) Visits and Log of Total ED Visits in 2020 and 2021 Relative to the Corresponding Baseline Levels in 2019

	**Change in log of ED visits due to MH relative to 2019**	**P Value**	**Change in log of total ED visits relative to 2019**	**P Value**
Weeks 1-11 in 2020	0.131	0.001	0.0538	0.01
	(0.0260)		(0.0172)	
Weeks 12-23 in 2020	-0.230	0.000	-0.389	0.000
	(0.0263)		(0.0316)	
Weeks 24-35 in 2020	-0.0302	0.36	-0.153	0.000
	(0.0315)		(0.0271)	
Weeks 36-47 in 2020	-0.0725	0.09	-0.142	0.000
	(0.0376)		(0.0129)	
Weeks 47-53 in 2020	-0.123	0.01	-0.177	0.000
	(0.0339)		(0.00337)	
Weeks 1-11 in 2021	-0.00341	0.93	-0.153	0.000
	(0.0364)		(0.0220)	
Weeks 12-23 in 2021	-0.0677	0.06	-0.0500	0.05
	(0.0312)		(0.0226)	
Weeks 24-35 in 2021	-0.106	0.003	0.0213	0.33
	(0.0262)		(0.0207)	
Weeks 36-47 in 2021	-0.170	0.000	-0.0370	0.004
	(0.0303)		(0.00976)	
Weeks 47-53 in 2021	-0.187	0.000	0.0119	0.27
	(0.0287)		(0.0101)	
N	1570		1570	
R-sq	0.991		0.996	

Robust standard errors clustered by region in parentheses. All Coefficients are relative to the corresponding weeks in 2019. For example, Weeks 1-11 in 2020 is relative to Weeks 1-11 in 2019. These specifications control for region-fixed effects, week-fixed effects, year fixed effects, where the baseline year is 2019.

© 2023 Villas-Boas S et al. *JAMA Network Open*.

eAppendix 2. Alternative Specification for eTable 2

Looking at the specification in logs in the first column with the header log (MH Visits), which smooths out data outliers and has a percent interpretation, we see that visits for mental health decreased significantly by approximately 23% ($\hat{\beta} = -0.23$, $p < 0.001$) in the 11 weeks following the pandemic declaration relative to the same weeks in 2019. In the second column with the header log (Total Visits), we see that ED visits decreased by approximately 39% ($\hat{\beta} = -0.389$, $p < 0.001$) in the eleven weeks immediately after the pandemic was declared in 2020 relative to the corresponding weeks in 2019.

© 2023 Villas-Boas S et al. *JAMA Network Open*.
