# Supplementary material for: Patterns of US Mental Health–Related Emergency Department Visits During the COVID-19 Pandemic
Source: JAMA Netw Open. 2023 Jul 11;6(7):e2322720. doi: 10.1001/jamanetworkopen.2023.22720 (PMC10336606; doi:10.1001/jamanetworkopen.2023.22720)
Supplement: Supplement 2. — Data Sharing Statement [file jamanetwopen-e2322720-s002.pdf]

Data Sharing Statement

Villas-Boas. Patterns of US Mental Health–Related Emergency Department Visits During the COVID-19 Pandemic. *JAMA Netw Open*. Published July 11, 2023. doi:10.1001/jamanetworkopen.2023.22720

Data
Data available: No

Explanation for why data are not available: The data cannot be shared but can be obtained by submitting a request to the National Syndromic Surveillance Program (NSSP), Division of Health Informatics and Surveillance (DHIS), in the Center for Surveillance, Epidemiology and Laboratory Services (CSELS), and the Centers for Disease Control and Prevention (CDC).
